# The role of artificial intelligence in advancing urologic care: From diagnostics to therapeutics

**DOI:** 10.1016/j.sipas.2025.100322

**Published:** 2025-11-29

**Authors:** Sina Samenezhad, Dorna Rafighi

**Affiliations:** a Urology Department, School of Medicine, Shahid Beheshti University of Medical Sciences, Tehran, , Iran; b Department of Microbiology, Faculty of Basic Sciences, Research Sciences Branch, Islamic Azad University, Tehran, Iran

**Keywords:** Artificial intelligence, Urology, Machine learning, oncology, BPH, Radiomics

## Abstract

Artificial intelligence (AI) is gradually altering urology by improving diagnostic precision, prognostic evaluation, and therapy decisions in a broad spectrum of urologic diseases. Utilizing machine learning, deep learning, and radiomics, applications of AI have exhibited promise in enhancing cancer identification, stratification, and therapy response prediction, especially in prostate, bladder, and kidney cancers. Beyond cancer therapy, AI enables individually tailored care for benign diseases like benign prostatic hyperplasia, urolithiasis, Functional Urology even in pediatrics by enhancing diagnostic ability and outcome prediction. Heterogeneity of data, model explainability, ethical issues, and lack of prospective validation constrain incorporation into everyday practice. This review summarizes current applications and discusses methodological and ethical limitation, and defines future directions toward enhancing multidisciplinary interaction, standardization across datasets, and prudent implementation. Eventually, AI provides large-scale opportunity to transform urologic care by facilitating individually tailored, expedient, and equitable patient care.

## 1 Introduction

Artificial Intelligence (AI) is described as computers that simulate human thought like learning, reasoning, and making decisions. In healthcare environments, AI by machine learning, deep learning, and associated algorithms facilitates enhanced diagnosis, support for treatment decisions, and clinical interventions. Processing high-dimensional data, AI enhances diagnostic accuracy, facilitates individualized treatments, and identifies patterns possibly invisible to humans [[1], [2], [3]].

AI has applications across all urological subspecialties, including improving diagnostic accuracy in uro-oncology, enhancing disease severity assessment in pediatric urology, and streamlining treatment decisions for benign prostatic hyperplasia (BPH), urolithiasis and functional Urology . For urolithiasis, it offers compositional analysis of stones and therapy planning support. For pediatric urology and BPH, it offers support for disease severity and surgical outcomes. For uro-oncology, it offers prognosis prediction, prediction of response to therapy, and recurrence monitoring through integration with imaging and genomic evaluation. Techniques such as radiomics and cystoscopy augmented by AI enhance cancer detection and grading, such as Gleason score. Functional urology also benefits from AI through decreased procedural intrusiveness and diagnostic accuracy. Through augmentation by augmented reality, AI has potential use in surgical teaching and simulation of patient variability. All these advances promote urological use of AI through increased clinical efficiency and customization of care to individuals [4,5].

Though exceptional advances have occurred, urological research involving AI lags disproportionately compared with cancer research and remains highly biased toward studying the prostate. Subspecialties such as BPH, pediatric urology, and functional urology are poorly represented. Methodologic flaws are characteristic with large numbers of studies employing small, homogeneous groups, often retrospective ones that restrict external validity. Other limitations include excessive use of binary classification models, poorly interpretable models, lack of explainability, and inadequate measures to compensate for algorithmic bias. Heterogeneity in imaging protocols and patient populations compromises model robustness. Ethic problems such as bias, privacy of data, and lack of explainability regarding how an AI program operates are not well addressed and serve as barriers to securing trust and adoption. For optimal realization of benefits from AI, future research must prioritize multicenter prospective studies along with standardized practices for data and development of robust ethical frameworks [[6], [7], [8], [9]].

This review aims to provide a general assessment of current uses, advantages, and constraints of AI in urology with special focus on its diagnosis, prognosis, and therapy in both oncologic and non-oncologic conditions. The review critically evaluates prevailing methodological trends, and focuses on essential issues such as data bias, ethical considerations, and explainability. Through synthesis of previous evidence and determination of future prospects, this review aims to guide clinicians, scientists, and policymakers toward responsible and effective integration of AI in urological practice.

## 2 Methodology

This narrative review synthesizes the current applications of artificial intelligence (AI) in urology across both oncological and non-oncological settings. A systematic approach was used to conduct the search in PubMed, Scopus, and Web of Science. These keywords included those like "artificial intelligence," "machine learning," "radiomics," as well as descriptive words like "prostate cancer," "bladder cancer," "renal cell carcinoma (RCC)," "pediatrics," "functional urology," "benign prostatic hyperplasia (BPH)," and "urolithiasis." Only publications that came out in the period between 2010 and January 2025 were taken into account. Emphasis was laid upon peer-reviewed journals as well as high-quality systematic reviews concerning the AI in urology in the domains of diagnostics, prognosis, treatment, as well as ethical matters. The study was not a systematic review at all, yet instead a narrative review, although a structured technique was adopted, no formal statistical examination was conducted.

The inclusion criteria were peer-reviewed articles and high-quality systematic reviews that touched upon the applications of AI in urology, particularly diagnosis, prognosis, treatment, and moral obligations. The most consideration was provided to the studies related to RCC, pediatric urology, functional urology, and BPH. The exclusion criteria were non-peer-reviewed articles, non-AI or non-urology related, and the studies that did not cover the main ideas of AI in the areas of diagnosis, prognosis, or treatment of urology.

It was conducted by two independent reviewers, who collaborated. Any disputes among the reviewers were resolved by consensus. No formal quality assessment was being undertaken although the methodological quality of all the studies included in the review was considered. The overall purpose was to evaluate the impact of AI on clinical decision-making, as well as patient outcomes, in urology, and to establish predominant trends, challenges, and future areas of research.

## 3 The impact of AI on cancer diagnostics

### 3.1 AI in prostate MRI interpretation

#### 3.1.1 AI enhancements in prostate MRI interpretation

Machine learning-based prostate cancer imaging, especially using convolutional neural networks (CNNs) and radiomics, enhances the interpretation of prostate MRI substantially. The software decreases inter- and intra-reader variation and provides more accurate risk stratification and the ability to detect lesions, beyond the accuracy of conventional PI-RADS. AI tools, such as CNNs, reduce false positives and enhance clinical decision-making, although challenges remain in ensuring external validation and high-quality datasets for broader application [[10], [11], [12]].

#### 3.1.2 Machine learning and radiomics for improved risk stratification

Radiomics-based machine learning algorithms enable enhanced risk stratification accuracy. For example, a logistic regression algorithm using radiomic variables was able to achieve higher AUC (0.870 vs. 0.658) in place of PI-RADS, with enhanced sensitivity, specificity, and predictive potential. AI-supported segmentation enhances the accuracy of the contours of the prostate as well as the lesions, with reduced processing time and enhanced clinical workflow, such as 3D model-guided prostatectomy [[10], [11], [12]].

#### 3.1.3 Integration of clinical guidelines into AI models

Recent advances have incorporated clinical guidelines directly into AI models. A multi-modal large language model (MLLM) integrating the Prostate Imaging Clinical Guidelines (PICG) with MRI data has enhanced diagnostic accuracy without requiring additional network parameters. This approach provides a more standardized decision-making framework and improves diagnostic consistency, particularly for indeterminate cases, with high precision in differentiating PI-RADS 3 lesions [13,14].

Lastly, AI facilitates improved interpretation of prostate MRI by minimizing inter-observer discrepancy, improved diagnosis accuracy beyond conventional PI-RADS grade levels and allowing more precise assessment of lesions with highest relevance in indeterminate cases. By incorporating the combination of radiomics, deep learning, and inclusion of clinical guideline, AI-based models exhibit higher reliability as well as generalizability across institutional settings.

### 3.2 AI in bladder cancer

#### 3.2.1 AI and imaging in bladder cancer diagnosis

Bladder cancer, amongst the most frequent malignancies, relies heavily on accurate staging in order to delineate muscle-invasive and non-muscle-invasive illness. Imaging, predominantly multiparametric magnetic resonance imaging (mpMRI), is the foundation of the process because it can provide high-resolution, functional, and anatomical data without the hazard of ionising radiation. The Vesical Imaging–Reporting and Data System (VI-RADS) was developed to standardize mpMRI acquisition and interpretation, ensuring reproducible muscle invasion assessments. However, its application is challenged in post-treatment scenarios due to anatomical distortions, which complicate image interpretation [15].

#### 3.2.2 AI's role in enhancing bladder cancer imaging

AI, specifically deep learning and radiomics, is revolutionizing bladder cancer imaging by the automation of the time-consuming process of tumor segmentation, thereby increasing the accuracy of diagnostics. AI models reduce observer-to-observer variability by a significant amount and lead to more consistent staging across muscle-invasive and non-muscle-invasive bladder cancer. AI-based radiomics models are also highly accurate in the prediction of treatment response, allowing for individualized treatment plans. However, the clinical application of AI in bladder cancer imaging is restricted by challenges, such as anatomical distortion in post-treatment imaging and the need for prospective validation [15].

#### 3.2.3 AI-Enhanced imaging biomarkers for tumor detection

AI-based quantitative imaging biomarkers, using T2-weighted, diffusion-weighted (DWI), and dynamic contrast-enhanced (DCE) MRI, show great promise in tumor subtyping, characterization, and detection. AI-based biomarkers significantly improve diagnosis by attenuating observer variability and shortening the imaging interpretation time. This AI-assisted process is critical in improving the overall quality of bladder cancer imaging and advancing diagnostic capabilities [16].

#### 3.2.4 Artificial intelligence and longitudinal therapy assessment

Artificial-intelligence-assisted radiomics is enabling the extraction of high-dimensional imaging characteristics, facilitating longitudinal assessments of these features through delta-radiomics. This approach provides a more refined measurement of therapy responses compared to conventional methods such as RECIST. If integrated with relevant clinical indicators, AI-driven models can offer precise therapy decisions and improve prognosis assessments, potentially enhancing personalized patient care [17].

#### 3.2.5 Future prospects and challenges

Even though AI in bladder cancer imaging is yet in its infancy, it has great potential for increasing the accuracy of diagnosis, the monitoring of therapy, and even the end result in patients. Yet, its clinical application remains hampered by some of the challenges such as post-therapy anatomical distortion and the lack of validation studies. Eradication of these challenges stands to bring big breaks in the diagnosis and treatment of bladder cancer [[15], [16], [17]].

### 3.3 AI in renal cancer

AI has become a transformative tool in the diagnosis and management of renal cell carcinoma (RCC). AI models leveraging imaging data, particularly through radiomics, allow for the precise differentiation between malignant and benign renal lesions, improving early detection and risk stratification. Machine learning models have demonstrated high sensitivity and specificity in RCC diagnosis, sometimes surpassing human clinicians in accuracy. However, challenges remain, such as the lack of prospective, multicenter studies to validate these models, as well as the issue of external verification. To achieve widespread clinical adoption, these models must be rigorously tested and standardized across various healthcare settings.

#### 3.3.1 Early detection and risk stratification

Early renal cell carcinoma (RCC) detection is essential in the improvement of patient survival, and AI algorithms exhibit promising accuracy in the differentiation of malignant and benign renal lesions. Meta-analyses pooled sensitivities ranging from 80 to 85 % and specificities ranging from 75 % to 90 %, often higher or equal to human clinicians, particularly in the aspect of specificity [18].

#### 3.3.2 Radiomics and imaging biomarkers

AI-augmented radiomics enables quantitative imaging biomarkers to be obtained from CT and MRI images so that detailed cancer characterization and prediction of biological behavior are possible. Such models refine risk stratification and permit non-invasive prediction to facilitate personalization of therapy. Additionally, integrative AI models that utilize imaging data with clinicopathologic features have significant promise for advanced and metastatic RCC care; such studies in this context are, however, insufficient [19,20].

#### 3.3.3 Challenges and future directions

Notwithstanding these developments, there are still several challenges. Problems like inter-study variation, external verification, and shortage of big-scale prospectively designed multicenter studies need to be overcome before AI models can gain widespread acceptance in daily practice. These hurdles need to be broken down in order for AI-based tools to reach their full potential in the diagnosis and management of RCC. When AI models are further standardized and validated, they can gain wide entry into routine practice for RCC and stand to improve diagnosis accuracy and prognosis [19,20].

## 4 AI in benign urological conditions

### 4.1 BPH

#### 4.1.1 Challenges in BPH diagnosis and treatment

Benign prostatic hyperplasia (BPH) is a common illness in aging men, frequently presenting lower urinary tract symptoms (LUTS) like frequency, urgency, and incomplete bladder emptying, heavily impacting the quality of life. Even though benign, the natural history of BPH is extremely variable, leaving the decision in favor of either medical therapy or surgery tricky. Artificial intelligence (AI) is increasingly an essential resource in managing these challenges by advancing diagnosis, predicting response to therapy, and individualizing treatment plans. AI programs process non-invasive information like ultrasound, uroflowmeter, and symptom scores, incorporating patient-specific variables to individually forecast responses to drug or operative therapy. This data-oriented practice encourages individualized medicine and encourages more patient-focused clinical decision-making. AI not only helps in the discrimination of BPH from other overlapping conditions like prostate cancer but has the potential to exceed in the optimization of BPH-specific diagnosis and care pathways, decreasing variability, and providing evidence-based care. However, more studies and validation are needed in order to ensure that the incorporation of AI is accurate, safe, cost-effective, and suitable in a wide range of clinical settings [[21], [22], [23]].

#### 4.1.2 AI in diagnostic precision for LUTS

AI has exhibited great potential in enhancing the diagnostic assessment of male LUTS related to BPH. Machine learning algorithms, especially convolutional neural networks (CNNs), evaluate uroflowmetry measurements and symptom scores in order to distinguish among LUTS causes, i.e., bladder outlet obstruction (BOO) and detrusor underactivity (DUA). AI-based models improve the accuracy of diagnosis, minimize diagnosis variability, and aid in more tailored treatment protocols for BPH. Importantly, AI is also involved in the prediction of drug responses so that clinicians can individualize therapies to the actual needs of individual patients. Yet, the external validation of these models is limited, and more studies are required to establish their clinical value in practical settings. For example, the researchers in one study applied a fine-tuned VGG16 CNN architecture to the evaluation of uroflowmetry graphs in over 1700 patients and exhibited moderate accuracy in the separation of DUA and BOO with area under the receiver operating characteristic (AUROC) indicators of 73.3 % and 72.2 %, respectively. Another study investigated the application of machine learning models, i.e., CatBoost and XGBoost, to databases containing voiding parameters, ultrasound characteristics, and patient complaints. XGBoost generated higher overall diagnosis accuracy than CatBoost with AUROC indicators of 0.826 for BOO and 0.819 for DUA, while CatBoost had higher sensitivity in screening, and XGBoost, higher specificity in diagnosis accuracy. These AI-based methodologies offer accurate, non-invasive, and affordable substitutes for conventional urodynamic studies, allowing for improved clinical decision-making and more suitable patient handling [24,25].

#### 4.1.3 Predicting therapy response and tailored treatment

AI also holds significant promise in the clinical management of BPH by improving diagnostic precision, predicting drug response, and supporting individually tailored treatment decisions. Various models, including fuzzy logic models, computer vision systems, and clinical data mining schemes, have demonstrated diagnostic accuracies of over 96.3 %. These models overcome the limitations of traditional measures like uroflowmetry and symptom scores by analyzing advanced clinical data to make more objective decisions. While the results are promising, AI’s current applications face challenges, such as variability among models and insufficient external validation, limiting generalizability. As development continues and AI is more widely integrated into clinical practice, these technologies are expected to refine BPH management, reduce diagnostic variability, and ultimately improve therapy outcomes [26].

### 4.2 Urolithiasis

#### 4.2.1 AI in detection of urinary calculi

Artificial intelligence has demonstrated profound capability in enhancing the detection and classification of urinary calculi, particularly through the analysis of imaging and intraoperative visual information. Deep learning networks, such as convolutional neural networks (CNNs) like ResNet, have been successfully applied to kidney–ureter–bladder (KUB) radiographs to attain over 97 % accuracy levels or better, with some models achieving impeccable specificity in diagnosing urolithiasis. Such computer-aided diagnosis (CAD) systems are particularly useful in emergency settings, facilitating non-specialist clinicians in image evaluation at skill levels comparable to expert radiologists [27].

#### 4.2.2 AI in predicting stone composition

Beyond initial detection proficiency, artificial intelligence has proved remarkable in anticipating the composition of stones via intraoperative endoscopic visual images. For instance, studies utilizing ResNet-101 analyzed >1600 high-quality images of stones extracted from 490 patients with precision levels for predicting composition between 98 % and 100 % across several classifications of stones, including those with dual compositions. Beyond that, AI-operated video classifiers trained on digital recordings from ureteroscopy successfully recognized morphological traits in real time with balanced precision amounting to 88 %. Such sophisticated technologies outdo human visual estimation and assist in concluding prompt intraoperative decisions such as changing holmium laser settings according to the projected composition of stones. Altogether, the AI-assisted investigation of static and dynamic visual inputs offers an impressively precise, time- and clinically relevant alternative to conventional methodologies of diagnosis [[27], [28], [29], [30]].

#### 4.2.3 Predicting kidney stone recurrence

Predicting recurrent kidney stones remains difficult given the multi-factorial etiology. Machine learning techniques are increasingly employed to process large volumes of complicated clinical data to predict patients at greater risk of recurrence and improve outcomes. A large cohort study involving >1200 patients had several machine learning models such as LASSO regression, random forest, and gradient boosted trees built from electronic health record data and results from 24-hour urine tests to estimate symmetric kidney stone recurrence at 2 and 5 years. Of these models, LASSO exhibited the best predictive ability by modestly outperforming conventional logistic regression. Important predictors consistently featured in patient age, BMI, and gout history.

In one small patient cohort undergoing endoscopic combined intrarenal surgery (ECIRS) for calcium calculi, one such deep learning model known as TabNet showed enhanced precision in predicting recurrence at two years. Feature explanation identified elevated urinary oxalate levels and modest postoperative reductions in hemoglobin levels, despite remaining normal, as relevant predictors. A lean version of the TabNet model incorporating only three variables the urine oxalate, urine volume, and postoperative hemoglobin changes retained robust predictive ability and was therefore applicable to clinical practice. These results put strong emphasis on building prediction models incorporating comprehensive urine chemistry in addition to perioperative data and imply that existing clinical best practices may fail to discriminate at-risk patients for recurrence. For kidney stone patients as a whole, machine learning holds out hope to refine stratification by personalization and optimize follow visits such that patient outcomes may be optimized [31,32].

### 4.3 Functional urology

#### 4.3.1 Advancements in diagnostic accuracy

The integration of Artificial Intelligence (AI) into functional urology is reshaping diagnostic and treatment paradigms. AI has shown promise in improving the accuracy of urodynamic studies (UDS), which were traditionally complex and invasive. Machine learning (ML) models, particularly neural networks (NN), can process uroflowmetry data with diagnostic accuracies exceeding 90 %, strongly correlating with invasive pressure-flow studies [33]. These advancements have the potential to reduce the need for invasive testing, improving patient comfort and clinical efficiency. Additionally, deep learning technologies applied to automated UDS data analysis enhance the detection of subtle patterns, improving the diagnosis of complex conditions such as detrusor overactivity and bladder outlet obstruction with sensitivities and specificities exceeding 80 % [34].

#### 4.3.2 AI in surgical education and training

AI is also enhancing surgical education in functional urology. By utilizing AI-driven metrics, such as automated performance recordings, surgical procedures, including bladder and prostate surgeries, can be analyzed for skill levels and improvements. This provides a systematic and efficient approach to advancing the education of urologists, promoting consistent skill development in functional urology procedures [34].

#### 4.3.3 Predicting treatment responses

AI is being increasingly applied in the prediction of treatment response for the diseases of functional urology. There are machine learning models that can predict treatment outcomes in diseases like benign prostatic hyperplasia (BPH) by the early prediction of the nonresponders in the treatment protocol. These AI models, based on the combination of the clinical, demographics, and imaging data, provide patient outcome predictions, like the risk of incontinence following prostatectomy [33]. There has also been promise in the application of AI in the treatment outcome prediction in overactive bladder (OAB), giving us tools that form customized and more precise plans for the management [34].

#### 4.3.4 Innovation in urology devices

AI is also being used to develop new devices to facilitate functional urology. Devices like the e-bladder diary, that, through AI, can track the urinary routine in real-time, allow the patient as well as the healthcare professional to track as well as take control of bladder function beyond the office. Others are electromechanical artificial urinary sphincters as well as remote monitoring devices, that can assist in the future of telemedicine as well as expand the reach, in particular, in the underprivileged or rural areas [34].

#### 4.3.5 AI's role in urodynamic study interpretation

Urodynamic studies, including cystometries and pressure-flow tests, are critical for diagnosing functional urological disorders but require specialized expertise. Recent studies have shown that AI can assist in interpreting urodynamic traces by learning standardized practices and terminology. Systems like ChatGPT, when taught using International Continence Society (ICS) guidelines, have shown improvement in interpreting urodynamic traces. This indicates that AI could support clinicians in educational settings and clinical decision-making, helping standardize interpretations and increasing access to expert-level analysis [34].

#### 4.3.6 Challenges and future directions

Despite these developments, challenges persist in the wide-scale implementation of AI in functional urology. The main issues are the requirements for big, standardized databases to enhance the generalizability and validation of AI models. Most studies so far have poor sample sizes, limited external validation, and the lack of multicenter studies, confining the widespread clinical implementation of AI. The future studies must build over these hurdles by increasing the sharing of data and multicenter studies to create more stable and scalable AI models [5,33,34].

Lastly, AI's place in functional urology is rapidly evolving, with much potential to expand diagnosis, treatment planning, and patient outcomes. As AI tools continue to increase integration in clinic operations, their potential benefit in diminishing diagnostic mistakes, customizing treatment plans, and increasing surgery training is likely to redefine the future of functional urology. However, addressing quality, standardization, and validation challenges in the data shall ensure that the innovations realize their full potential in practice.

## 5 The role of artificial intelligence in pediatric urology

Artificial intelligence (AI) and machine learning (ML) are revolutionizing pediatric urology, providing improved diagnostic preciseness, individualized treatment, and enhanced disease management. Yet, as much as AI-based applications in pediatric urology are increasing, much is still problematic in the arena of validation, clinical integration, and quality of data.

### 5.1 Advancements in diagnostic accuracy

AI is having an increasing role in pediatric urology diagnosis. There is an increasing application of machine learning models to classify conditions such as hydronephrosis, VUR, and UTIs. AI enhances the accuracy of diagnosis in imaging, allowing measurement of the severity of conditions such as hydronephrosis and posterior urethral valves [35,36]. One study demonstrated that AI could classify uroflowmetry curves with 85.8 % accuracy, reducing observer variability and improving diagnoses [37].

### 5.2 Predicting treatment outcomes

AI has the potential as well in the prognosis of pediatric urology cases. Data-based models, such as imaging and urodynamic studies, assist in the prediction of the outcome of treatment for certain cases like VUR and hydronephrosis. Through the determination of the chance of success or failure in surgeries, AI supports clinicians in the choice of the best treatment [36,38]. This personalized approach can help optimize care and reduce unnecessary procedures.

### 5.3 Enhancing disease management

AI is being applied to the treatment of chronic pediatric urological diseases. Using longitudinal data, AI models can forecast the trajectory of diseases like neurogenic bladder and kidney impairment. Through the examination of longitudinal data, AI models can identify patients at risk early enough, allowing timely intervention [39]. Additionally, AI-powered remote monitoring tools can track urinary function in children with chronic conditions, offering real-time insights and improving long-term care [36].

### 5.4 Addressing the AI chasm

Despite promising results, AI models face limitations due to the "AI chasm" the gap between models developed in research settings and their real-world application. Most models have been validated in small, single-institution datasets, limiting their generalizability. Pediatric urology's unique challenges, such as low-frequency conditions and complex phenotypes, further complicate AI model development. Overcoming these barriers requires large-scale, multicenter collaborations and standardized datasets [35].

### 5.5 Future directions

The AI in Pediatric Urology (AI-PEDURO) collaborative is working to bridge these gaps by creating an online repository of AI models. This platform will help clinicians assess the validity of models and encourage collaboration for further validation [35]. Future efforts should focus on improving model interpretability and ensuring that AI tools adhere to ethical standards for clinical use [39].

## 6 AI in robotic surgery and intraoperative guidance

Artificial intelligence is increasingly playing a pivotal role in robotic-assisted surgery by facilitating skill evaluation, identification of surgical maneuvers, and intraoperative support. AI systems involving standardized objective metrics (SOMs), e.g., OSATS and GEARS, have been utilized to assess surgeon proficiency with moderate to nearly perfect reported accuracies. Despite this, large variability in reliability remains evident in numerous studies. More advanced AI models involving convolutional neural networks (CNNs) in conjunction with temporal scrutiny through long short-term memory (LSTM) layers automatically learn surgical maneuvers from videos with very high precision. Additionally, these models effectively differentiate surgeon skill levels with promising objective measures throughout surgeon learning and potential real-time applications throughout intraoperative stages. Additional models like SATR-DL utilize parallel deep learning networks to quantify raw motion data in spite of collection throughout robotic operations, facilitating accurate evaluation of trainee proficiency as well as identification of intended surgical activities, e.g., suturing and knot-tying. Extracting advanced spatial and temporal characteristics directly from motion sequences, this data-driven approach transcends conventional manual evaluation to enhance both efficiency and accuracy in surgical skill assessment [[40], [41], [42]].

In combination, these artificial intelligence technologies are able to redefine intraoperative guidance by automating skill evaluation and yielding actionable comments. Advances like these have the potential to enhance surgical education programs and ultimately enhance operative outcomes.

## 7 AI in decision support and patient monitoring

Artificial intelligence (AI) is increasingly employed to improve patient care in surgical disease and chronic disease. AI-powered predictive models have been demonstrated to be more accurate than traditional risk calculators in identification of patients at highest postoperative complications risk. Enhanced predictive capability facilitates more appropriate preoperative stratification by risk and allows for personalization of informed consent discussions [43].

Besides surgical care, other AI tools such as mobile apps, reminder systems, and patient empowerment platforms are utilized to enhance medication adherence among individuals with non-communicable diseases. The technologies encourage patient-healthcare provider communication and medication use monitoring alongside integrated care models, all in bids to ultimately garner larger adherence rates and improved clinical results. Implementation of these AI solutions is only successful if strong data infrastructure is in place and if diversity among users is carefully considered to prevent exacerbation of healthcare inequality. Additional research must justify these tools among diverse patient groups and in varied clinical settings, especially in view of pandemic-facilitated accelerated implementation of digital health technologies to counteract COVID-19 [44].

While AI is beginning to automate and standardise administrative and clinical work flows in out-patient urology, wide-spread clinical integration is delayed by such problems as adaptation of workflow and stringent proof prior to wider adoption [45]. As shown in Table 1, AI applications span various urological domains, including oncological and benign conditions.

**Table 1:** Summary of AI Applications and Benefits across Urological Conditions and Cancer Types.

Condition/Cancer Type	AI Application	Benefits
Prostate Cancer	MRI Interpretation, CNNs for Lesion Detection, Multimodal Integration (AI + Clinical Guidelines)	Improves detection accuracy, reduces false positives, enhances risk stratification, and lesion detection; reduces inter-reader variability; enhances decision-making for PI-RADS 3 lesions
Bladder Cancer	Image Analysis (mpMRI, VI-RADS), Tumor Segmentation, Radiomics, AI-driven Biomarkers (T2, DWI, DCE MRI), Delta-Radiomics	Enhances tumor detection, staging consistency, reduces observer variability, helps predict treatment response, improves therapy monitoring, enables personalized care
Renal Cancer	Lesion Characterization, Radiomics, Imaging Biomarkers, AI Radiomics Models for Biological Behavior Prediction, Multi-modal Data Integration	Differentiates malignant from benign lesions, improves early detection and risk stratification, non-invasive prediction for advanced/metastatic RCC
Other Cancers	Pathology Analysis, Tumor Classification, AI-driven Risk Assessment, Immunotherapy Prediction	Supports diagnostics, improves tumor subtyping, enhances treatment decision-making, aids in immunotherapy planning
Benign Prostatic Hyperplasia (BPH)	Symptom Analysis, Uroflowmetry Data, AI Algorithms for Treatment Prediction, Predicting Drug Response	Improves diagnostic precision, predicts therapy responses, personalizes treatment, reduces variability in outcomes, reduces misdiagnosis with prostate cancer
Urolithiasis (Kidney Stones)	Imaging (KUB, CT scans), Stone Detection, Predicting Recurrence, Urinary Chemistry Models for Recurrence	Enhances detection and classification of stones, aids in treatment planning, predicts recurrence risk, tailors follow-up care, optimizes management of recurrent stones
Functional Urology	Urodynamic Studies and Automated Analysis, Surgical Skill Assessment (AI Metrics), Predicting Treatment Outcomes, Urodynamic Trace Interpretation	Improves diagnostic accuracy, enhances surgical training, personalizes treatment plans, reduces complications, reduces unnecessary invasive testing, enhances decision-making for bladder outlet obstruction and detrusor overactivity
Pediatric Urology	Diagnostic Imaging, Machine Learning for Condition Classification (Hydronephrosis, VUR), Predicting Surgical Success, AI Models for Treatment Outcomes	Enhances diagnostic precision for pediatric conditions, improves early diagnosis, predicts treatment success, improves personalized care and management of chronic conditions

## 8 Ethical, practical, and technical challenges

The incorporation of AI in healthcare, and urology in particular, presents major ethical challenges that need to be handled delicately to provide fair, patient-focused care. One of the most important challenges is reducing algorithmic bias emanating from non-representative datasets. If biased or homogenous AI models are trained, they risk reinforcing or even amplifying the current healthcare inequalities. This is especially true for under-represented groups or in low- and middle-income countries (LMICs), where healthcare information is less robust or diverse. Fairness in algorithm design and the use of diverse, representative datasets is needed for AI instruments to perform in various populations and settings. In addition, resolving the issues of data privacy and confidentiality is very important, as AI systems generally demand massive volumes of sensitive healthcare information. Guaranteeing strong data governance and the conscious patient consent models is needed, whereby patients can make conscious decisions about how their information is used, especially in the context of AI training and clinical implementation [46].

### 8.1 Transparency and explainability

Another basic ethical issue is the "black box" status of most AI models, which decreases transparency and thus trust and responsibility. To bring AI models into practice successfully, models need to be explainable so that clinicians as well as patients can understand how decisions are made. Transparency leads to trust and indicates that AI acts as an ancillary, not a substitute, for human decision-making. Ongoing ethical surveillance is required to uncover unintended consequences and make tools for AI a benefit to all populations equally [46].

### 8.2 Regulatory frameworks and clinical adoption

regulatory policies governing the application of AI in healthcare. These involve the following: Adhering to the directives of the regulatory institutions like the FDA and the EMA, responsible for governing AI medical devices; The EU AI Act, responsible for laying down ethical requirements and safety criteria for the application of AI in healthcare. Knowledge of these policies is critical in making AI tools conform to set safety measures and being AI-ready for use in the clinic. In addition, models that are generated in high-income settings tend to lose their applicability when generalized in the LMIC settings because of the disparities in healthcare infrastructure, the nature of patients, and available databases. Transfer learning as well as fine-tune AI systems to local-specific conditions can tackle these challenges [47,48].

### 8.3 Clinician-Centered decision-making

In spite of the rising hope surrounding tools of AI, clinicians shall continue to form the kernel of decision-making in the care of patients. The available AI technologies yet lack full convergence with human clinical thinking, making them less useful in practical fields. In an attempt to mitigate the gap, AI systems can be made to enhance the decision-making of clinicians by being flexible to the decision-making practices of clinicians. This can be done by obtaining behavioral information of clinicians in order to hone AI algorithms, so that they prove supportive as opposed to invasive in the clinical practice [49]. AI needs to serve as the assistant to the clinicians, making them better at making accurate, informed decisions as opposed to substituting in the decision-making process [49].

By addressing these ethical, practical, and regulatory concerns, we can ensure that AI tools in urology are ethically sound, reliable, and capable of delivering equitable, patient-centered care.

## 9 Future directions

The future of AI in urology will require focused efforts to overcome existing challenges and harness its full potential [1,6–9,50]. Key areas for improvement include:

### 9.1 Multicenter prospective validation studies

AI models must undergo validation in multiple populations and practice sites in clinical settings in an attempt to increase their external validation and generalizability. Large-scale multicenter studies are essential in the confirmation of the usefulness and reliability of AI-based approaches in clinical practice, especially for under-represented diseases such as pediatric and functional urology.

### 9.2 Federated learning for data sharing

Federated learning models can be considered in order to respond to the issues of data sharing and privacy. The decentralized models of AI enable training based on distributed databases without sharing patient information, while allowing broader collaboration of the data by preserving the privacy, particularly in low- and middle-income countries (LMICs).

### 9.3 Incorporation into clinical trials

Integration of AI models in clinical trials can provide robust evidence of their safety and efficacy in real-world situations. These shall narrow down the applications of AI and make their integration in clinical practices more streamlined, leading to improved patient outcomes and treatment plans.

### 9.4 Standardization of AI models and protocols

Another essential step is the formulation of standardized procedures for AI model development, data, and reporting. This shall help in increasing reproducibility, reducing bias, and increasing the accuracy and transparency of models, so as to gain higher trust and acceptance among patients and clinicians.

### 9.5 Ethical and regulatory advancements

For AI to integrate easily into clinical practice, there must be sustained efforts in addressing ethical challenges such as algorithmic bias, explainability, and transparency. There must be adherence to regulations like the FDA, the EMA, and the EU AI Act so that AI tools can meet the required safety and ethical thresholds in healthcare. By filling these gaps, future studies can make AI models more accurate, fair, and clinically efficient in the delivery of urological care, and overall enhance decision-making and patient outcomes

By addressing these gaps, future research can ensure AI models become more accurate, equitable, and clinically effective in urological care, ultimately improving decision-making and patient outcomes

## 10 Conclusion

Artificial intelligence set to revolutionize urological landscape by enhanced diagnostic and prognostic certainty and improved therapy choices in a wide spectrum of urological afflictions ranging from urological malignancies to benign diseases like BPH and urolithiasis. Computer-aided technology in the form of sophisticated imaging procesing, predicting models, and intraoperative localisation systems offer unrivalled opportunities of maximizing patient benefits as well as clinician convenience. Urological research under AI remains in nascent form and is hampered by non-generalizability due to heterogeneity in the data, lack of explainability and bias, and inadequacy of appropriate multicentre validations.

In realizing the potential of artificial intelligence in urology, future efforts must place utmost priority on generating large, heterogeneous, and standardized datasets, performing interdisciplinary endeavors, and integrating ethical models for fair and responsible deployment.. Clinicians will always have to be at the center of the technology-enabled care pathway and will need education and engagement to close loops between technology and clinician judgment. Through sustained innovation and rigorous testing, artificial intelligence will emerge as a normalized component of urological care specific to individuals and ultimately improve patient outcomes and chart the future of the specialty

## Ethical statement

This article is a narrative review based on previously published studies and does not involve any original research with human participants or animals. Therefore, ethical approval and informed consent were not required.

## Consent for publication

Not applicable. This review article does not contain any individual person’s data in any form (including individual details, images, or videos).

## CRediT authorship contribution statement

**Sina Samenezhad:** Writing – review & editing, Writing – original draft, Validation, Supervision, Methodology, Funding acquisition, Formal analysis, Conceptualization. **Dorna Rafighi:** Writing – original draft, Visualization, Validation, Project administration, Methodology, Investigation, Conceptualization.

## Declaration of competing interest

The authors declare that they have no known competing financial interests or personal relationships that could have appeared to influence the work reported in this paper.

## Data Availability

Data availability is not applicable to this article.
